# Relationship of screen time with anxiety, depression, and sleep quality among adolescents: a cross-sectional study

**DOI:** 10.3389/fpubh.2024.1459952

**Published:** 2024-11-29

**Authors:** Nur Zakiah Mohd Saat, Siti Aishah Hanawi, Hazlenah Hanafiah, Mahadir Ahmad, Nor M. F. Farah, Nur Ain Atikah Abdul Rahman

**Affiliations:** ^1^Biomedical Science Program, Faculty of Health Sciences, Centre for Community Health Studies (ReaCH), Universiti Kebangsaan Malaysia, Kuala Lumpur, Malaysia; ^2^SOFTAM, Faculty of Information Science and Technology, Universiti Kebangsaan Malaysia, Bangi, Malaysia; ^3^College of Computing, Informatics and Mathematics, Universiti Teknologi MARA Sabah Branch, Kota Kinabalu Campus, Kota Kinabalu, Malaysia; ^4^Clinical Psychology and Behavioural Health Program, Faculty of Health Sciences, Center for Community Health Studies (ReACH), Universiti Kebangsaan Malaysia, Kuala Lumpur, Malaysia; ^5^Faculty of Health Sciences, Center for Community Health Studies (ReaCH), Universiti Kebangsaan Malaysia, Kuala Lumpur, Malaysia

**Keywords:** adolescent, anxiety, depression, screen time, sleep quality

## Abstract

**Introduction:**

In the current digital age, people’s use of electronic devices has significantly increased screen time, which may have an impact on different aspects of their lives. Adolescents today are exposed to excessive screen time, which may affect their sleep and contribute to anxiety and depression. The purpose of this study is to examine the relationship between screen time with sleep quality, anxiety and depression, among adolescents in Klang Valley, Malaysia.

**Methods:**

This study is a cross-sectional study information was gathered from among 353 secondary school students in the Klang Valley using a questionnaire. The instrument that was used in this study was Pittsburgh Sleep Quality Index (PSQI) Malay version, screen-based media usage (SCREENS-Q) and Hopkins Symptom Check List-25 (HSCL-25) Malay version. The sampling method was stratified and convenience sampling method. The analysis study used the Smart Partial least squares (PLS) method to analyze the data.

**Results:**

Using the Smart PLS technique, we examined the relationship between these variables and identified revealed that screen time has a direct, positive, and significant impact on anxiety level (Mean = 0.134, *β* = 0.123, *p* < 0.01) and depression levels (Mean = 0.202, *β* = 0.194, *p* < 0.01). Moreover, screen time has a low effect on sleep quality (Mean = 0.128, *β* = 0.117, *p* < 0.05). However, the mediating factor, sleep quality, was not significant in the indirect effect of screen time with anxiety (Mean = 0.047, *β* = 0.040, *p * > 0.05) and depression (Mean = 0.044, *β* = 0.043, *p*  < 0.05).

**Discussion:**

This study highlights the importance of understanding the association between screen use, sleep quality, anxiety and depression. Notably, excessive screen time appears to be associated with poorer sleep quality, ultimately increasing anxiety and depression. Understanding the effects of excessive screen time on sleep and well-being may have a substantial impact on public health policies and interventions. Enacting policies that promote better screen habits and sleep hygiene could improve people’s overall quality of life and well-being in the digital age. However, more longitudinal research is needed to confirm the causality of these relationships and investigate potential intervention strategies.

## 1 Introduction

In today’s digital age, the term “screen time” has become increasingly prevalent, referring to the amount of time an individual spends in front of a screen, including devices such as smartphones, computers, televisions, and tablets. As technology has become an integral part of daily life, screen time has expanded beyond leisure and entertainment to encompass work, education, and social interaction. While screen time offers numerous benefits, such as instant access to information, connectivity, and entertainment, there is growing concern about its potential effects on health and well-being. There are no specific guidelines regarding the acceptable amount of screen time for adolescents. The earlier recommendation from the American Pediatric Association was to limit screen time to 2 h per day (1). However, previous studies have categorized screen time among adolescents into two ranges: low screen time, defined as less than 2 h per day, and excessive screen time, defined as more than 4 h per day (2).

Quality of life (QOL) reflects a person’s or group’s overall well-being across various aspects of life, capturing both positive and negative factors at a given point in time (1). Key elements influencing QOL include personal health, work environment, relationships, and financial stability. QOL measurements help assess an individual’s experience of health and illness, offering insight into how to improve life quality. Important variables affecting QOL encompass physical and mental health, education, personal growth, social support, economic status, and personal values (2). A Canadian study found a significant positive relationship between screen time and levels of anxiety and depression among students, particularly with computer use and video gaming, but not with television viewing (3). A previous study found an association between screen time and anxiety. A longitudinal study of adolescents in the United Kingdom found that the risk of anxiety was higher among adolescents who spent 3 h on the computer during weekdays and weekends compared to 1 h per day, respectively (4). Depression among adolescents has also been associated with social media. For example, a systematic review showed that there was a significant relationship between social media use and depression (5). According to a different study on the association between screen time and psychological well-being, adolescents between the ages of 14 and 17 who spent an average of 7 h a day in front of a screen were more likely to experience symptoms of depression and anxiety. In addition, those who used screens more frequently (7 h) than those who used them less frequently (1 h) were twice as likely to procrastinate, struggled to maintain composure, and argued frequently with carers (6). In addition, a study conducted on Danish adolescents revealed conflicting results regarding the relationship between screen time, that is, the amount of time spent on computers and television, and depression severity. Meanwhile, an association was found between the amount of time adolescents spent watching television and their symptoms of depression on the major depression inventory (MDI). Nevertheless, there was no discernible association found between computer use and depression (7).

Adolescents and young adults between the ages of 13 and 18 need adequate sleep to support their physical and mental health and to facilitate a healthy transition into adulthood. Teenagers who have difficulty getting enough sleep are more likely to experience depression, insulin resistance, diabetes, hyperactivity, impaired memory, decreased learning, and lower grades (8, 9). As a result, poor sleep quality can negatively affect multiple areas of a person’s life, leading to a decline in their overall quality of life. A previous study of adolescents found a moderate association between screen time and both poor academic performance and reduced sleep quality. According to the study, weekday screen time among adolescents aged 9 to 10 was not associated with depression or anxiety, but it was associated with poor sleep quality, duration, and academic performance (10). Research involving college students revealed that a significant percentage of them experience poor sleep quality (11–13). Although students understand how important it is to get the right amount and quality of sleep, few of them make it a habit (14).

Adolescents who use screens for over 4 h daily, such as for video games, are more likely to face sleep difficulties than those who use them for less than 1 h (15). Studies show that excessive computer and television use can negatively impact sleep quality (16, 17). Internationally, many teenagers experience sleep deprivation on weekdays due to high levels of screen time, which can affect academic performance and overall health (18). Notably, using media devices before bedtime can lead to sleep deprivation, with Internet addiction being a significant factor. Research highlights that excessive screen time, particularly in the evening, harms sleep quality by interfering with melatonin production due to blue light exposure (19, 20). Teenagers often use electronics late at night, disrupting their sleep schedules, and younger individuals are more likely to use devices right before bed, contributing to insufficient rest (13.22).

A study of high school students in other countries found that 74% reported experiencing poor sleep quality during the COVID-19 pandemic, as assessed by the Pittsburgh Sleep Quality Index (PSQI) questionnaire (21). According to the PSQI, a score above 5 indicates poor sleep quality (21). Enhancing the overall quality of life of adolescents requires improving the quality of their sleep. Rising screen time among various age groups, particularly adolescents, has increased concerns among parents and educators. At the same time, there has been a notable rise in anxiety and depression among adolescents. Previous studies indicate that increased screen time, especially before bedtime, may disrupt sleep and lead to poor sleep quality, which may be associated with elevated levels of anxiety and depression. The purpose of this study is to determine the relationship between screen time with sleep quality, anxiety and depression.

## 2 Materials and methods

### 2.1 Study design and sampling

This research employed a cross-sectional design, with data collection conducted from September 2021 to April 2022 in the Klang Valley, Selangor, which is located in the central region of Peninsular Malaysia. Participants were recruited using a convenience sampling method; parental consent and participant consent were obtained prior to data collection. The Ministry of Education Malaysia provided a list of schools, and the researchers selected schools through stratified random sampling, utilizing a random table for selection. The target population included all high school adolescents from grades 1 to 5 in the Klang Valley. The sample size was determined using Cochran’s formula (1977) (22) with a prevalence of poor sleep quality from a previous study (*p* = 0.24) (23) and a margin of error of ∆ = 0.046. The formula used was $n=\frac{p\left(1-p\right)z^{2}}{\Delta^{2}}\text{.}$ Inclusion criteria were adolescents aged 13–18 years who owned digital devices such as smartphones, laptops, or desktop computers and had received informed consent from their parents to participate. Exclusion criteria included adolescents taking medication for mental health conditions or those diagnosed with sleep disorders, such as insomnia.

The included adolescents were given the option to either accept or reject taking part in the survey, and their participation was voluntary. In addition, parental consent was obtained for all participants under the age of 18 years. The study followed ethical guidelines to ensure the well-being and privacy of the participants throughout the research process. The Ethics Committee of Universiti Kebangsaan Malaysia has approved this study with reference code JEP-2021-503. The questionnaire was printed and distributed to the participants.

In this study, one parent, either the mother or father, completed a questionnaire about screen time using an adapted version of the SCREEN-Q (24). At the same time, the adolescents filled out a questionnaire evaluating anxiety, depression, and sleep quality. Parents of adolescents aged 13–17 years were included in the study. Parents with mental health issues that interfered with their ability to answer the questionnaire were excluded from the study.

The schools were chosen from a list of Klang Valley institutions using stratified random sampling, with stratification based on age group. Students from the selected schools were then chosen using a convenience sampling method.

### 2.2 Measures

This study used a questionnaire to measure three main variables: demographic questions, screen time, quality of sleep, quality of life, anxiety, and depression. The demographic questions were about the name of the school, age, and gender. The Screen-Q was utilized to evaluate the screen time question. The Screen-Q includes six domains: screen media environment, children’s screen media use, context of screen media use, early exposure, parental perceptions of screen media use, and parental media use (15). The six domains of the Screen-Q range exhibit reliability scores ranging from 0.67 to 0.90 across all domains. In this study, the researcher used the validated version of the Screen-Q, which was translated into Malay and tested for reliability and validity. The results of the validity test based on the content validity index from 10 experts showed that I-CVI was 0.75–1.00 and S-CVI/Ave was 0.97. Meanwhile, the reliability test score based on the intraclass correlation coefficient was 0.62–0.89, indicating good reliability. There are no specific scores in the Screen-Q to indicate excessive screen time. However, for this study, information such as screen time was used in the analysis. The parents filled out the Screen-Q questionnaire (24).

The Pittsburgh Sleep Quality Index (PSQI-M) for Malaysian populations (25) and the Hopkins Symptoms Checklist-25 (HSCL-25) were the tools used by the researchers to gage both anxiety and depression (26). The Pittsburgh Sleep Quality Index (PSQI) was used in this study to evaluate sleep quality. The internal consistency coefficient was found to be 0.74 (25). Overall, 19 items in the PSQI evaluate various aspects of sleep, including subjective quality, duration, habitual sleep behavior, sleep disturbances, latency, use of sleep medications, and dysfunction during the day. The overall PSQI score can range between 0 and 21, with each component having a score between 0 and 3. A score of 5 or less denotes very good sleep quality, and a score of more than 5 denotes bad sleep quality.

The Hopkins Symptoms Checklist-25 (HSCL-25) is a 25-item tool designed to measure the symptoms of anxiety and depression, impacting the overall quality of life (27). It is particularly suitable for adolescents and has been validated in Indonesia (28). The HSCL-25’s brevity and simplicity make it widely used across various populations, with translations available in several languages, including Sudanese, Arabic, French, and Indonesian (29–31). Research supports its validity and reliability, with an internal consistency coefficient exceeding 0.7. The checklist features two subscales: 10 items for anxiety and 15 for depression, rated on a 4-point scale from “Not at all” to “Extremely.” It has also been compared favorably to other assessment tools, such as the Beck Depression Inventory (BDI-II) and the Center for Epidemiologic Studies Depression Scale (CESD) (32, 33).

### 2.3 Statistical analysis

In this study, data analysis was conducted using IBM SPSS Statistics software version 27 and partial least squares structural equation modeling (PLS-SEM). PLS-SEM, which was performed using smart partial least squares (Smart PLS) version 4, was, therefore, the primary data analysis technique to test the study’s proposed hypotheses. The model presented in this study will feature observable variables, or indicators, of the suggested model, shown as rectangles, and latent variables, or constructs, represented as circles. Arrows between these elements. Researchers used these representations to build and estimate complex models through PLS-SEM (34, 35). Estimating the correlation between the latent variables was utilized to assess how well the model captured the target constructs of interest (36).

## 3 Results

### 3.1 Demographics

This study included 353 participants in total, representing all secondary school students and adolescents in Selangor. Of these, 37.4% were men, and 62.6% were women. The distribution of the socio-demographic analysis of the respondents who participated in this study is displayed in Table 1. A total of 353 school adolescents enrolled in secondary schools across three Klang Valley districts, ranging in grade levels from first to fifth, comprised the subjects of the study. Regarding the age group, 8.2% of the teenagers enrolled in Form 1 schools made up 11% of the respondents, followed by those in Form 2 schools, 6.8% in Form 3 schools, 18.4% in Form 4 schools, and the majority of the respondents, or 55.5%, were in Form 5 schools.

**Table 1:** Demographic factors and number of devices at home using descriptive statistics.

Variable		*n* (%)
Total sample		*n* = 353
Gender	Male	132 (37.4%)
	Female	221 (62.6%)
Age group	13	29 (8.2%)
	14	39 (11.0%)
	15	24 (6.8%)
	16	65 (18.4%)
	17	196 (55.5%)
House location	Rural area	106 (30.0%)
	Urban area	247 (70.0%)
Household income	Below RM1000	95 (26.9%)
	RM1001–RM3000	163 (46.2%)
	RM3001–RM6000	66 (18.7%)
	RM6001 and above	29 (8.2%)
Number of telephones	0	6 (1.7%)
	1	252 (71.4%)
	2	47 (13.3%)
	3	15 (4.2%)
	4	10 (2.8%)
	5	18 (5.1%)
	6	5 (1.4%)
Number of televisions	0	5 (1.4%)
	1	302 (85.6%)
	2	39 (11.0%)
	3	5 (1.4%)
	4	0 (0.0%)
	5	2 (0.6%)
Number of laptop	0	143 (40.5%)
	1	167 (47.3%)
	2	33 (9.3%)
	3	7 (2.0%)
	4	3 (0.8%)
Number of others device (Tablet, iPad, Xbox, PlayStation, Desktop)	0	287 (81.3%)
	1	53 (15.0%)
	2	12 (3.4%)
	3	0 (0.0%)
	4	1 (0.3%)

Furthermore, as shown in Table 1, regarding the number of devices owned, the typical respondent possessed a phone, television, laptop, or other electronic device. In the phone category, most respondents (13.7%, *n* = 47) reported owning two phones; similarly, a majority (11%, *n* = 39) of the respondents reported that they owned two televisions. Overall, 41% of the respondents did not own a laptop, while 47.3% (*n* = 167) reported having a laptop. In other device categories, such as tablets and video game consoles, 81.3% (*n* = 287) of the respondents stated they had none, whereas 15% (*n* = 53) reported owning at least one of these items. The average daily time spent on each device, along with any data spread or variation, is displayed in Table 2. The average and standard deviation for different devices provide information about how long participants spend using each particular device. Participants used their smartphones for 7 h per day on average. Meanwhile, as shown in Table 3, 58% (*n* = 205) of the respondents reported poor sleep quality. Regarding depression levels, 68.8% (*n* = 243) of the respondents reported no symptoms, while 67.4% (*n* = 238) of the respondents reported no symptoms of anxiety.

**Table 2:** Descriptive statistics.

Devices	Hours per day (Mean ± SD)
Smart phone	7.20 ± 3.814
Television	1.96 ± 1.877
Tablet/iPad	0.43 ± 1.214
Laptop	1.08 ± 2.349
Desktop	0.32 ± 1.014
Game console without grip (e.g., Xbox, PlayStation, Nintendo)	0.27 ± 0.841
Handheld game console (e.g., PSVita, PSP, Nintendo Switch, Gameboy)	0.17 ± 0.792
E-book	1.17 ± 1.856

**Table 3:** The descriptive results of sleep quality, physical activity, anxiety, and depression category.

Variable		Frequency	Percentage
Sleep	Good	148	42%
	Poor	205	58%
HSCL (depression)	No symptom	243	68.8%
	Has symptom	110	31.2%
HSCL (anxiety)	No symptom	238	67.4%
	Has symptom	115	32.6

### 3.2 Evaluation discriminant and convergent validity (outer measurement model)

Screen time, sleep quality, anxiety, and depression indicators were used in the measurement model in the first iteration of the path model. The model for screen time, however, showed four indicators; for sleep quality, it showed three indicators; and for anxiety and depression, it showed three indicators. The model was chosen for outer loading greater than 0.6, as suggested by earlier research (37). After removing the indicator with a value of less than 0.6, the model was utilized for additional analysis.

A number of criteria were evaluated to confirm the validity and reliability of the study’s measurement model, also referred to as the “outer model,” as suggested by previous researchers (38). These included “discriminant validity,” “convergent validity,” “composite reliability” (CR), and “internal consistency reliability” (Cronbach’s alpha). The study scale exhibited a satisfactory degree of internal reliability, as evidenced by Cronbach’s alpha (*α*) values, which ranged from 0.406 to 0.783 (39). In addition, the composite reliability (CR) values fell between 0.647 and 0.873, indicating that the internal consistency of the measurement model was sufficient.

**Figure 1:**
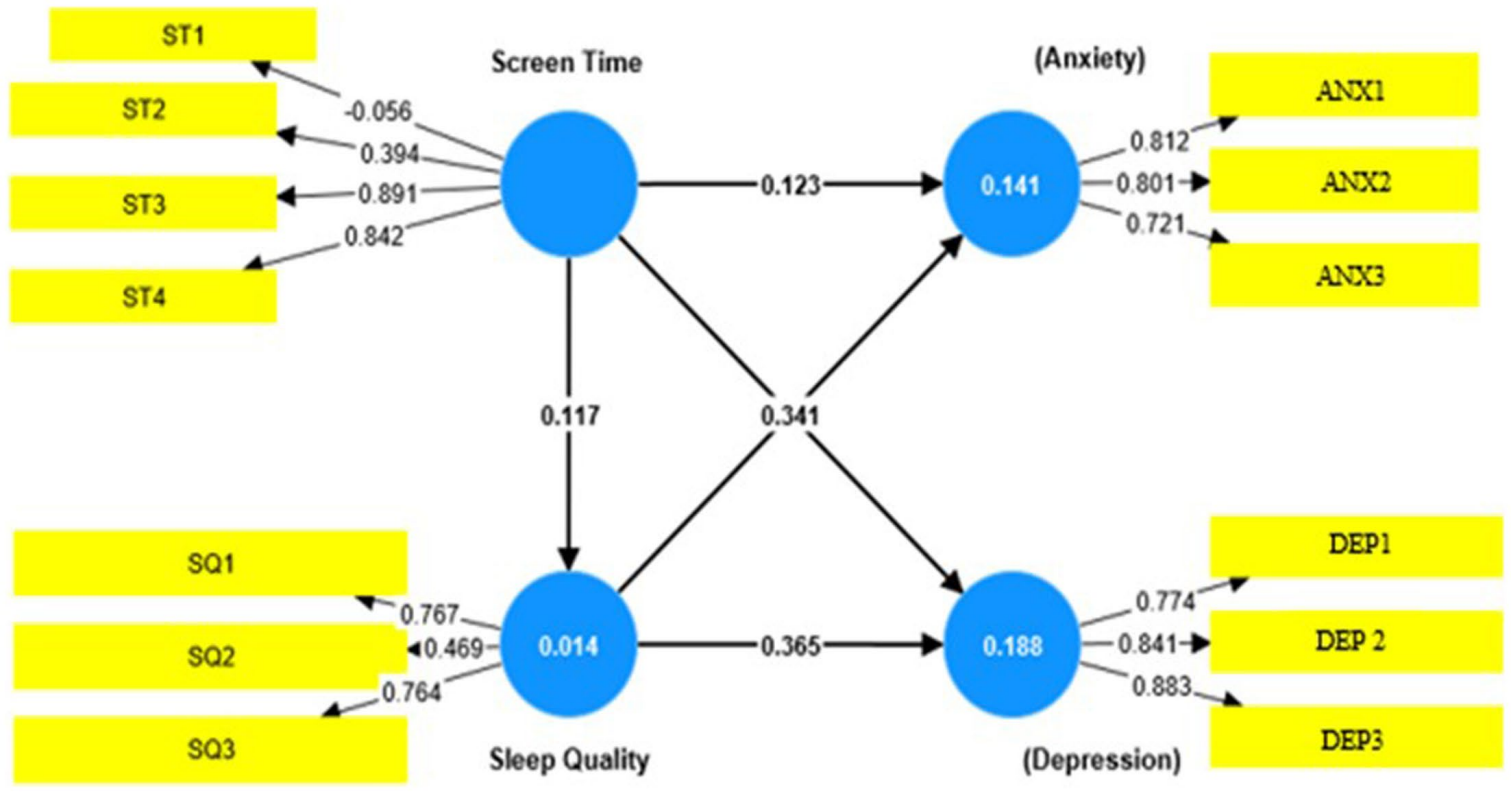
Structural and measurement model. ST1–ST4 refering to the question of screen time; SQ1–SQ3 referring to question in sleep quality; ANX1–ANX3 referring to the question of anxiety; DEP1–DEP3 referring to the question of depression.

Based on Figure 1, the indicators of screen time that were included in the model were ST1 “Total weekday e-reader screen time,” ST2 “Total weekday laptop screen time,” ST3 “Total weekday mobile phone screen time,” and ST4 “Total weekday mobile phone screen time.” Meanwhile, the indicators for sleep quality that were included in the model were SQ1 “In the past month, how often did you experience sleep problems because you have insomnia?,” and SQ2 “In the past month, how often did you experience sleep problems because you have nocturia?,” and SQ3 “In the past month, how often did you experience sleep problems because you are unable to fall asleep?.” Furthermore, the indicators for anxiety were ANX1 “In the past month, how often did you experience symptoms of anxiety (Feeling anxious)?,” ANX2 “In the past month, how often did you experience symptoms of anxiety (Feeling headaches)?,” and ANX3 “In the past month, how often did you experience symptoms of anxiety (Feeling afraid)?.” Moreover, the indicators for depression were DEP1 “In the past month, how often did you experience symptoms of depression (Crying easily)?,” DEP2 “In the past month, how often did you experience symptoms of depression (Feeling lonely)?,” and DEP3 “In the past month, how often did you experience symptoms of depression (Self-blaming)?”

There are two components to the path model. The first is the measurement model, also known as the outer model, which explains how the latent variables relate to their indicators. The second component is the structural model, which explains the relationships between the latent variables (40). Additionally, all quality of life-related factors, as well as factors ST3, ST4, SQ1, SQ3, and others, had “Standardized Factor Loading” (SFL) values greater than 0.70. This result provided more evidence in favor of the study’s dimensions and demonstrated a respectable degree of acceptable reliability. Nevertheless, it was significant that ST1 had negative standardized loading values, which suggests that these indicators and their corresponding latent constructs have a different relationship than what was first anticipated. To understand the underlying causes of this unexpected directionality and to evaluate the efficacy of these indicators in measuring the corresponding constructs, more investigation was therefore judged as necessary.

Furthermore, standardized loading values below 0.70 for some indicators (ST2 and SQ2) indicated a relatively weaker association with their latent constructs. Therefore, a thorough analysis was necessary to determine the reasons behind this weaker relationship and to assess the validity and reliability of these indicators. Subsequently, the assessment of convergent validity was performed by checking whether the average variance extracted (AVE) values exceeded 0.5, which served as the minimally acceptable level for confirming convergent validity (41). Previous research recommended considering composite reliability (CR) for AVE values less than 0.5. Convergent validity was established if the CR value was greater than 0.6. All of the CRs in this study were greater than 0.6 (42–44).

Three additional criteria, as proposed by previous researchers, were used to ensure the discriminant validity of the scale (45). The “heterotrait–monotrait method ratio” (HTMT), the “cross-loading matrix,” and the “Fornell–Larcker criterion method” were among these criteria. The following observations were made to ascertain the discriminant validity of the model. Table 4 shows that to guarantee discriminant validity, each variable’s outer loading (bolded) had to be greater than its cross-loading (with other measurements). Table 5 shows a model with a high degree of discriminant validity as indicated by the bolded diagonal. The average variance extracted (AVE) values exceeded the inter-variable correlation coefficient (42–46). It is recommended that HTMT values be less than 0.90. The HTMT values in this investigation were mostly significantly lower than the recommended threshold, as shown in Table 5, with the exception of one value which was 0.880. However, further analysis was performed using a bootstrap, and it was found that the confidence interval was less than 1, indicating that the discriminant validity was established (21). Similarly, these findings support the reliability, convergent validity, and discriminant validity of the scale—all of which were confirmed in the external measurement model of the study. Thus, the study can proceed with the structural outer model to test its hypothesis.

**Table 4:** Cross-loading for study factors.

	Screen time	Sleep quality	Anxiety	Depression
ST1	**−0.056**	−0.027	0.027	−0.059
ST2	**0.394**	0.014	0.096	0.145
ST3	**0.891**	0.103	0.137	0.206
ST4	**0.842**	0.118	0.130	0.162
SQ1	0.104	**0.767**	0.240	0.313
SQ2	0.064	**0.469**	0.118	0.163
SQ3	0.072	**0.764**	0.324	0.292
**Anxiety**				
ANX1	0.103	0.250	**0.812**	0.519
ANX 2	0.136	0.340	**0.801**	0.530
ANX 3	0.141	0.215	**0.721**	0.458
**Depression**				
DEP1	0.144	0.264	0.534	**0.774**
DEP2	0.241	0.332	0.529	**0.841**
DEP3	0.198	0.364	0.565	**0.883**

ST1–ST4 referring to the question of screen time; SQ1–SQ3 referring to question in sleep quality; ANX1–ANX3 referring to the question of anxiety; DEP1–DEP3 referring to the question of depression.

**Table 5:** Fornell-Larker criterion and HTMT results.

Fornell-Larcker criterion					HTMT results			
	1	2	3	4	1	2	3	4
1. Anxiety	**0.779**							
2. Depression	0.648	**0.834**			0.880			
3. Screen time	0.163	0.237	**0.644**		0.302	0.420		
4. Sleep quality	0.355	0.388	0.117	**0.681**	0.592	0.637	0.264	

### 3.3 Assessment of the study hypothesis (structural inner model)

In addition to examining how well the model explained and predicted variations in the endogenous variables brought on by the exogenous variable, structural equation modeling was used to test the study’s proposed hypotheses. A number of suggested criteria were used to evaluate the goodness of fit of the model, including the minimum *R*^2^ value of 0.10, the “Stone-Geisser Q2” of greater than 0.0, the normed fit index (NFI) of greater than 0.90, and the SRMR value of less than 0.08 (42). Table 6 illustrates that all endogenous dimensions, specifically the quality of life (anxiety) (*R*^2^ = 0.141, *Q*^2^ = 0.012), quality of life (depression) (*R*^2^ = 0.188, *Q*^2^ = 0.039), and sleep quality (*R*^2^ = 0.014, *Q*^2^ = 0.003), exhibited sufficient R2 and Q2 values. Within the model, the highest proportion of variation was depression (18.8%), followed by anxiety (14.1%) and sleep quality (1.4%). These findings suggest that the model accurately depicts the empirical data and has adequate predictive power (46).

**Table 6:** Coefficient of determination (*R*^2^) and (*Q*^2^) and model fit (SRMR-NFI).

Endogenous latent factors	(*R*^2^)	(*Q*^2^)
(Anxiety)	0.141	0.012
(Depression)	0.188	0.039
Sleep quality	0.014	0.003
**Model fit**	**SRMR**	**NFI**
	0.084	0.650

Finally, as shown in Table 7, a bootstrapping method in smart PLS4 was used to find the path coefficient and corresponding *t*-value for both direct and mediating interrelationships. Smart PLS results showed that screen time has a direct, positive, and significant moderate effect on anxiety (*β* = 0.194, *t*-value = 3.134, *p* < 0.01), a moderate effect on depression (β = 0.365, *t*-value = 8.052, *p* < 0.01), and a low effect on sleep quality (β = 0.117, *t*-value = 1.977, *p* < 0.05). In this study, there was a significant relationship between screen time and sleep quality, with indicators of experiencing sleep problems due to insomnia, nocturia, and difficulty falling asleep at night. Concurrently, there is a substantial significant strong effect between sleep quality and anxiety (β = 0.194, *t*-value = 7.051, *p* < 0.01) and a strong effect with depression (β = 0.365, *t*-value = 8.052, *p* < 0.01).

**Table 7:** Study hypothesis results.

Hypotheses	Mean	Beta (β)	(*T*-value)	*p* values	Results
Screen time → sleep quality	0.128	0.117	1.977	0.048	Supported
Screen time → anxiety	0.134	0.123	2.197	0.028	Supported
Screen time → depression	0.202	0.194	3.134	0.002	Supported
Sleep quality → anxiety	0.344	0.194	7.051	<0.001	Supported
Sleep quality → depression	0.368	0.365	8.052	<0.01	Supported
Screen time → sleep quality → quality of life (depression)	0.047	0.023	1.875	>0.05	Not Supported
Screen time →sleep quality → quality of life (anxiety)	0.044	0.022	1.833	>0.05	Not Supported

As shown in Table 7, the highest path coefficients were between sleep quality and anxiety (0.344) and sleep quality and depression (0.368). As shown in Figure 1, the direct hypotheses were supported, and the mediating effect of the sleep quality hypothesis was rejected. The results show that the effect size of the hypothesis was between 0.101 and 0.168, indicating that there was a medium effect size (21, 46).

## 4 Discussion

In the era of digital technology, screens of all kinds—from computers and televisions to smartphones and tablets—have become increasingly prominent in our daily lives. Although these devices have surely improved connectivity and convenience, excessive screen time has sparked worries about possible negative effects on several facets of our health. The association between screen time and overall quality of life and the quality of sleep is one important area of research (47, 48).

The study results showed that adolescents’ daily smartphone use averaged 7.2 hours, which is higher than findings from a previous study. Before the COVID-19 lockdown, adolescents spent an average of 4 hours per day on their smartphones, which increased slightly to 4.5 hours per day after the lockdown (49). Another study during the peak of COVID-19 also showed almost similar average hours using smartphones, which is more than 6 h per day (50). This indicates that the need to use smartphones during the post-COVID-19 pandemic among adolescents was high due to the necessity to do school assignments, and discussions were carried out mostly online, by using WhatsApp, Telegram, and other social media.

The results of this study indicated that 58% of the participants experienced poor sleep quality, which is higher than the 40% reported in a previous study (51). In addition, the findings demonstrated a significant relationship between screen time and sleep quality, suggesting that increased screen time is associated with lower sleep quality among adolescents. Sleep quality scores were determined based on a scale, with scores above 5 indicating poor sleep quality. Numerous studies have consistently highlighted the adverse effects of excessive screen time on sleep quality (3, 5). Increased screen time among adolescents due to digital devices has negatively affected sleep quality. The blue light from phones, tablets, and computers can suppress melatonin production, making it harder to fall asleep (52). A study found that adolescents aged 16–19 who met physical activity and screen time recommendations had a 73% lower chance of poor sleep quality (53). Evening screen use disrupts circadian rhythms, leading to difficulty falling asleep, shorter sleep duration, and fragmented sleep. Participants who slept near screens had a higher likelihood of poor sleep than those without screens in their environment. Engaging with stimulating digital content further raises cognitive arousal, complicating relaxation before bedtime, and ultimately worsening sleep quality (54, 55). The findings of this study are consistent with earlier research, showing that poorer sleep quality is associated with increased screen time. A study conducted in other countries used a cohort research design to examine the correlation between adolescent screen time and sleep quality. In that study, participants downloaded the software used to measure their screen time onto their mobile devices. The PSQI was used to gage the quality of the sleep. According to the findings, screen time and sleep quality are positively correlated, meaning that the more screen time, the lower the quality of sleep (50). The potential reduction in sleep duration is a significant concern associated with an increase in screen time. Digital devices often keep people awake late into the night, disrupting sleep. A study of Spanish adolescents found a significant association between screen time and cognitive inattentiveness, but not memory (56). Research on 9- to 10-year-olds showed that increased screen time during the weekdays was associated with attention issues, conduct disorders, and rule-breaking tendencies in both genders (10). Conversely, another study of youth aged 11–15 years reported that higher screen time positively affected working memory in male individuals (57). In addition to its influence on sleep, excessive screen time has broader implications for mental and physical health, thus affecting overall quality of life. In this study, the results also showed that screen time has a significant relationship with anxiety and depression levels. The level of significance was much higher in the relationship between screen time and depression using the HSCL-25. This study aligns with a previous study that found that there was a significant relationship between the duration of screen time, and anxiety and depression, controlling for gender, age, ethnicity, parental education, geographic area, physical activity, and body mass index (BMI) (58). There was relatively stronger evidence for associations between mobile phones and computers/Internet and subsequent depression (59, 60). The results of this study paralleled those of different age groups, such as younger age groups. For example, the study reported that among different age groups, such as young children and infants, television screen time had a low negative impact on cognition (61).

Furthermore, a significant association was found between screen time in young children (under 2 years of age) of less than 1 h and more than 1 h per day and behavioral problems, including aggression, anxiety, defiance, and others. The research design was a cohort study, and the behavior problems were assessed using the instrument Brief Infant-Toddler Social and Emotional Assessment (BITSEA) (62). A longitudinal design was used in a different study to discover a positive correlation between adolescent screen time and psychological issues (63). Another study, which used the Clinical Interview Schedule to assess anxiety and depression in adolescents in United Kingdom as part of a prospective cohort, found that computer use on both weekdays and weekends poses a slight risk of anxiety and depression (3).

The results of this study indicated a significant relationship between sleep quality and the levels of anxiety and depression, demonstrating a high level of significance. The findings are parallel to those of previous studies, in which depression was found to be closely associated with sleep disturbances, with many individuals experiencing insomnia or hypersomnia. Changes in sleep architecture, such as reduced REM sleep, are common in individuals with depression (64). Poor sleep quality can lead to increased feelings of fatigue, irritability, and cognitive impairment, further worsening depressive symptoms and reducing the individual’s capacity to cope with daily challenges. Individuals with anxiety disorders frequently report poor sleep quality, characterized by difficulties in falling asleep, staying asleep, and experiencing restful sleep. Anxiety often leads to hyperarousal, where individuals may have racing thoughts or excessive worry, making it challenging to relax and initiate sleep. This lack of quality sleep can then heighten anxiety symptoms, creating a vicious cycle (65).

The results of this study indicated that sleep quality was not a significant factor in mediating the relationship between screen time and anxiety and depression. The results contrast with a previous study conducted in China among adolescents, which found that sleep quality had a small effect in mediating the relationship between screen time and anxiety and depression, potentially contributing to increased anxiety and depressive symptoms (66). In addition, based on the review by previous researchers, there was a very small effect of the duration of screen time on depression and anxiety, as it depended on the type of device being used by the subjects. The most significant association between screen time and depression was observed among those who spent more time on mobile phones, computers, and the Internet. However, other forms of screen time, such as television, video games, and social media, had a smaller effect on depression and anxiety (53). Parallel to this study were the findings from another study about screen time and mental health problems in adolescents. However, the research used two different instruments to measure mental health: the Rosenberg Self-Esteem Scale, which measures adolescents’ self-esteem, and the Brief Symptom Inventory, which measures depression using randomized control trials (5).

In addition, the length of screen time was associated with anxiety and depression in another study. However, the study used two different instruments: the Multidimensional Anxiety Scale for Children to measure anxiety and the Children’s Depression Inventory for measuring depression using a cross-sectional study (67). Meanwhile, a different study conducted in New England discovered that adolescents’ depression could be measured using the Patient Health Questionnaire and Generalized Anxiety Disorder, respectively. A previous study found that substance use was significantly predicted by depression, anxiety, and increased screen time (66).

Previous studies have linked disrupted sleep patterns resulting from prolonged screen exposure to heightened stress levels, mood disorders, decreased psychological well-being, and poor lifestyle choices. The cognitive stimulation of digital content may contribute to feelings of restlessness and anxiety, further impacting mental health. In the study, it was indicated that in addition to screen time, lifestyle factors such as practicing a healthy lifestyle would also influence mood depression in subjects. Therefore, in addition to screen time and sleep duration, eating healthy foods can reduce and improve depression and anxiety levels in adolescents (67).

One key aspect of anxiety and depression is the impact of screen time on cognitive performance. Prolonged screen use can disrupt the brain’s transition from an active to a calm state, particularly before bedtime. Given that many adolescents rely on screens for virtual learning, this issue is especially relevant. They may connect with friends via apps such as WhatsApp or participate in virtual meetings through platforms such as Google Meet and Zoom. In addition, teachers often post announcements and facilitate learning via messaging services such as WhatsApp. Adolescents in this era are already accustomed to using the Internet to find information, particularly for assignments, group projects, and other purposes. As a result, they must use devices such as laptops, smartphones, and others to complete their assigned work. Naturally, this has led to a greater amount of time spent on these devices. Lack of sleep can negatively impact memory consolidation, cognitive function, and general productivity. However, adolescents can significantly improve their quality of life by finding a balance between screen time, sleep, and socializing with friends and family (67–71).

In conclusion, the association of screen time with effects on sleep quality, anxiety, and depression is imperative due to the ubiquitous influence of screens in our daily lives. In the digital age, maintaining a healthy balance between technology use and restful sleep habits is crucial to reducing these negative impacts and fostering a comprehensive sense of well-being. Future research and public health programs will investigate ways to promote a balanced approach to screen time, physical activity, and good sleep hygiene.

## 5 Limitations and implications

This study has several limitations. First, the relationship between screen time, sleep quality, and quality of life was explored using a cross-sectional research design, which limited our ability to identify causal effects. In addition, the research was conducted only in Klang Valley and a few schools in Selangor, limiting the applicability of the findings to other cities. Furthermore, the study focussed exclusively on adolescents, which means it does not represent a broader population. Another limitation is the self-reported nature of the questionnaire; the information on screen time was provided by parents discussing it with their children, while the assessments of anxiety and depression were completed by the adolescents themselves, which may have led to response bias.

The findings of this study may guide public health interventions and educational initiatives that seek to improve individuals’ standard of living and quality of life by encouraging healthy screen time and sleep patterns. More research is necessary to investigate the underlying mechanisms and potential moderating factors that contribute to the observed relationships.

## 6 Conclusion

Overall, the findings add to our understanding of how screen time and anxiety and depression are related, with sleep quality not appearing as a significant mediating factor. However, there is a significant direct relationship between anxiety, depression, and the quality of sleep. More research is required to explore potential other mediating factors and gain a deeper understanding of the basic mechanisms underlying these relationships.

## Data Availability

The original contributions presented in the study are included in the article/supplementary material, further inquiries can be directed to the corresponding author.
